# Thermal Stability, Dynamic Mechanical Analysis and Flammability Properties of Woven Kenaf/Polyester-Reinforced Polylactic Acid Hybrid Laminated Composites

**DOI:** 10.3390/polym14132690

**Published:** 2022-06-30

**Authors:** M. N. M. Azlin, S. M. Sapuan, M. Y. M. Zuhri, E. S. Zainudin, R. A. Ilyas

**Affiliations:** 1 Laboratory of Biocomposite Technology, Institute of Tropical Forestry and Forest Products, Universiti Putra Malaysia, Serdang 43400, Selangor, Malaysia; mohdazlin@uitm.edu.my (M.N.M.A.); zuhri@upm.edu.my (M.Y.M.Z.); edisyam@upm.edu.my (E.S.Z.); 2 School of Industrial Technology, Department of Textile Technology, Universiti Teknologi MARA, Cawangan Negeri Sembilan, Kampus Kuala Pilah, Kuala Pilah 72000, Negeri Sembilan, Malaysia; 3 Advanced Engineering Materials and Composites Research Centre, Department of Mechanical and Manufacturing Engineering, Universiti Putra Malaysia, Serdang 43400, Selangor, Malaysia; 4 School of Chemical and Energy Engineering, Faculty of Engineering, Universiti Teknologi Malaysia, Johor Bahru 81310, Johor, Malaysia; ahmadilyas@utm.my; 5 Centre for Advanced Composite Materials (CACM), Faculty of Engineering, Universiti Teknologi Malaysia, Johor Bahru 81310, Johor, Malaysia

**Keywords:** woven kenaf, woven polyester, polylactic acid, DMA, thermal, flammability

## Abstract

This paper presents the thermal and flammability properties of woven kenaf/polyester-reinforced polylactic acid hybrid laminated composites. The effects of the fiber content and stacking sequences of hybrid composites were examined. The hybrid composites were fabricated using the hot press method. Thermogravimetric analysis, differential scanning calorimetry, dynamic mechanical analysis, and flammability properties of woven kenaf/polyester-reinforced polylactic hybrid composites were reported. The thermal results have demonstrated the effect of the hybridization of the composites on the thermal stability and viscoelastic properties of the laminates. The work also measured the burning rate of the hybrid composites during the flammability test. The S7 sample that consisted of all woven kenaf layers in composite recorded the highest char residue of 10%, and the S8 sample displayed the highest decomposition temperature among all samples. However, as for hybrid composites, the S5 sample shows the optimum result with a high char yield and exhibited the lowest burning rate at 29 mm/min. The S5 sample also shows the optimum viscoelastic properties such as storage and loss modulus among hybrid composites.

## 1. Introduction

Fiber-reinforced polymer composites (FRPC) consist of either natural, synthetic, or a combination of both fibers to reinforce the polymer matrix. The selection of fibers and matrix is very important as the properties of the composites will greatly depend on the properties of individual materials for the products [1]. The increasing environmental awareness regarding pollution and waste management also has driven the industry to shift its attention toward bio-based materials, which are environmentally friendly and are much cheaper [2].

Natural fibers such as hemp [3,4,5], pineapple leaf [6,7], flax [8], oil palm [9], and sugar palm [10,11] have been used in composite manufacturing. Moreover, natural fibers in a woven form such as bamboo [12,13], jute [14,15], kenaf [16,17], and banana [18] also have been extensively used in composite fabrication. Alavudeen et al. [19] compared different forms of fiber between woven and short fiber for banana/kenaf polyester composites in terms of mechanical properties for the same composition. The result revealed that the woven banana/kenaf polyester hybrid composite has better mechanical properties due to the presence of fibers in continuous form. The incorporation of natural fibers into the polymeric system will affect the properties of the fiber polymer composites. However, using natural fibers only can be unsuitable for some products; thus, synthetic fibers are still used in composite manufacturing [20].

The environmental concern also has driven the emergence of natural-based polymers to be used in composite manufacturing. Recently, polylactic acid (PLA) has appeared as one of the most important polymers that has been used as a matrix in composite manufacturing [21]. PLA also has been commercially substituted for some petroleum-based polymers in various applications and is readily available in the market at a price that is comparable with other polymers such as polypropylene [22]. Despite the availability of natural fibers and environmental issues, synthetic fiber such as polyester is still being widely used as a material to produce composites [11,23,24]. Its durability, cheap cost, and ability to withstand moisture are the main reasons why polyester is still the material of choice in composite manufacturing [25,26,27].

The thermal behavior of the different materials in hybrid composites can be further understood using thermogravimetric analysis, differential scanning calorimetry, and dynamic mechanical analysis. The data obtained from these tests can reveal the thermal characteristics and behavior of the composites. The thermal properties of the hybrid composites depend on various factors such as fiber content, type of fibers, matrix, and manufacturing process [28]. Sathyaseelan et al. [29] studied the effect of stacking sequences of woven areca/kenaf-reinforced epoxy composite on the dynamic mechanical analysis. The author concluded that woven kenaf in the outer layer of the hybrid composites had revealed a balanced behavior between glassy and rubbery regions. Moreover, Oliveira et al. [30] concluded that the increased woven fique fiber content had raised the storage modulus and loss modulus of the composites.

Fiber-reinforced polymer composites have also been used in various areas such as marine [31], aerospace, construction, automotive, medicine, and sports [32,33]. As most application areas of composites may deal with heat and fire, the thermal and flammability behavior of the composites needs to be taken into consideration. The thermal and flammability properties of the composites are essential parameters that can sometimes restrict the application of the composites, especially in the construction and automotive area [34]. Hence, the later problem associated with thermal degradation and flammability behavior of the composites could be predicted. In aerospace applications, for instance, the composites are required to be lightweight as the composites will be used in manufacturing the structure of the plane (tails, wings, and fuselage) and must also be strong enough to withstand the high loads [35]. Moreover, despite the aerospace industry, the lightweight properties of the composites are required in the automotive industry to meet the fuel efficiency requirement [36]. However, most of the previous works on woven kenaf hybrid composites focused on investigating the mechanical performance of the hybrid composites [16,37,38,39,40,41], and only a few [42,43,44] studied the flammability and thermal properties of the woven composites.

Based on the literature, it was found that there is no research on the thermal and flammability properties of woven kenaf/polyester/PLA hybrid composites. Previous literature also suggested that the optimum fiber loading for hybrid composites is 40 wt% [45,46]. In this study, the woven kenaf and woven polyester were stacked in different layers and fiber content with a control fiber weight of 40%. The research aims at evaluating the effect of different stacking sequences and fiber content of woven kenaf/polyester/PLA hybrid composites on the thermal and flammability properties of hybrid laminated composites. Six hybrid laminate composites were fabricated, and two reference laminates of woven kenaf/PLA and woven polyester/PLA were also fabricated.

## 2. Materials and Methods

### 2.1. Material

The 100% woven kenaf and 100% woven polyester were used as reinforcement for polylactic acid (PLA) to fabricate the hybrid composites. Woven kenaf (WK) was purchased from Acheh, Indonesia, and woven polyester (WP) was supplied by Composites Ltd. in Staffordshire, UK. As for the matrix (PLA), it was supplied by Shanghai Huiang Industrial Co. Ltd. in Shanghai, China. The kenaf fiber composition is presented in Table 1.

### 2.2. Material Preparation

Woven kenaf, woven polyester, and PLA were prepared with the dimension of 200 × 200 mm. Woven kenaf and woven polyester were oven-dried at 60 °C for 24 h to remove moisture before the composite fabrication process.

### 2.3. Fabrication of Composite Laminates

**Figure 1:**
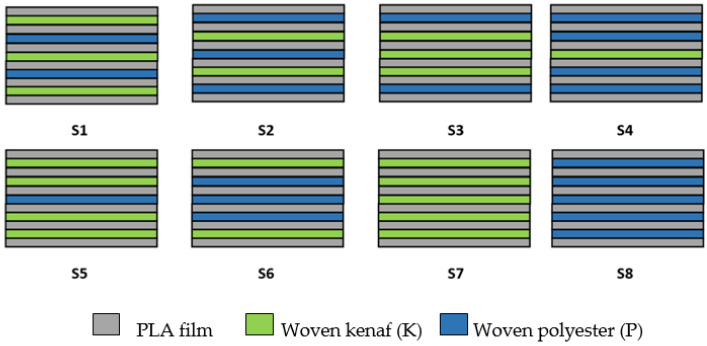
Stacking sequences and fiber content of laminated composites.

The hybrid composites were produced using a film-stacking method with five layers of fabrics (kenaf/polyester), and six layers of PLA film were stacked alternately in a mold, as shown in Figure 1.

The surface of the mold was previously sprayed with a mold release agent to prevent the matrix from sticking to the mold after the hot compression process. The kenaf and polyester fabrics were stacked in the same direction (warp/weft) with different stacking sequences and then were compressed using Vechno Vation (40 tonnes) hot compression machine. The PLA film softened and conformed to the shape of the mold. The fiber-to-matrix (%wt.) ratio was prepared with a fixed ratio of 40:60, and the stacking configuration for each laminated composite is presented in Table 2.

The hot compression plates were heated from room temperature until the temperature reached 165 °C. The stacked material in a mold was placed between the previously heated plates before being compressed. The layered material between the plates was further pre-heated at 165 °C for 10 min. After pre-heating, the plates were closed, and the samples were compressed at 165 °C for another 10 min. The sample was later cold pressed for another 5 min before being taken out immediately after the time ended.

### 2.4. Thermogravimetric Analysis (TGA)

The thermal degradation behavior of the hybrid composites was evaluated using a Q500 thermal analyzer (Washington, DC, USA). The sample was drilled to form a hole, and the drilled sample was collected up to a weight of 5 mg and placed in the thermal analyzer sample compartment. The samples were heated up to 600 °C of temperature under a nitrogen atmosphere with a 10 °C/min heating rate. The weight of the samples gradually decreased with the increment of temperature.

### 2.5. Differential Scanning Calorimetry (DSC)

A thermogravimetric instrument model Q500 (Washington, USA) was used to perform the differential scanning calorimetry tests. The testing temperature was gradually increased in the range of 25–300 °C at a heating rate of 10 °C/min. Several peaks can be observed in the thermogram to indicate the glass transition temperature (T_g_), melting temperature (T_m_), and cold crystallization temperature (T_c_) of the hybrid composites.

### 2.6. Dynamic Mechanical Analysis (DMA)

The DMA properties of the laminated composites were evaluated using a Perkin Elmer D8000 DMA Analyzer. Samples in the dimension of 10 mm in width, 30 mm in length, and 3 mm in thickness were prepared. The composites were tested from a temperature range of 30–150 °C, at an oscillation frequency of 1 Hz and a heating rate of 5 °C/min.

### 2.7. Flammability (UL-94)

The flammability test (UL-94) was carried out in accordance with ASTM International D635 [47]. The samples in the dimension of 125 × 13 × 3 mm were clamped horizontally at one end, and a burner was placed toward the other end for the flame to impinge on the free end. The time and extent of burning were measured for the flame that traveled from the 25 mm marking to 100 mm needed to be recorded. The burning rates of the composites were calculated as follows (Equation (1)) [48,49,50]:

V = 60L/t(1)

where V is the burning rate (mm/min), L is burned length (mm), and t is the time of burning (sec).

## 3. Results

### 3.1. Thermogravimetric Analysis (TGA)

**Figure 2:**
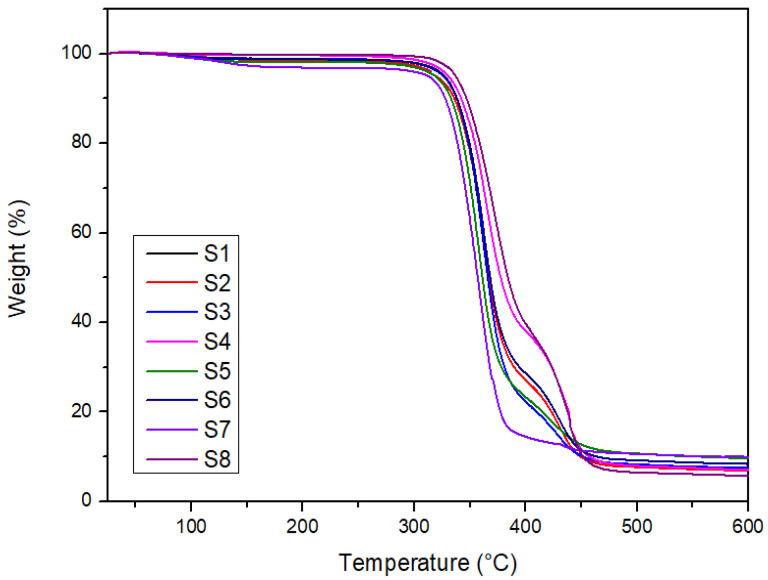
TG thermogram of laminated composites.

**Figure 3:**
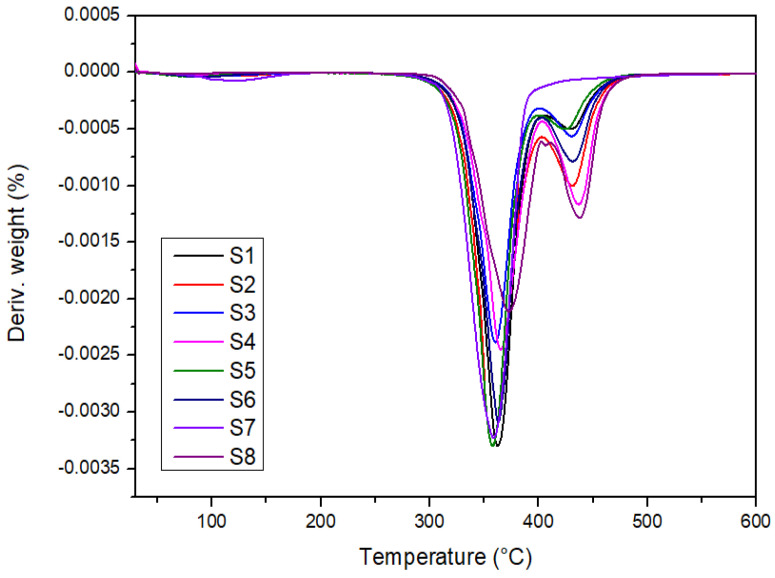
DTG thermogram of laminated composites.

Figure 2 and Figure 3 show the thermogram of weight loss as a function of temperature for the laminated composites (S1–S8). The degradation of the samples occurred within the temperature range of 25–600 °C. The degradation stages such as initial degradation, major degradation, final degradation, and char amount can be observed based on the TG and DTG thermogram. Figure 2 revealed three steps in the thermal degradation of the laminated composites.

The small initial degradation step can be observed below 100 °C. The weight loss of the sample at this stage is due to the loss of moisture and dehydration of the samples [51,52,53]. The moisture release and dehydration of woven kenaf in the temperature range of 30–110 °C contributed to the weight loss. The main thermal degradation stage can be observed in the temperature range of 250–370 °C. The degradation of three major constituents of natural hemicellulose in woven kenaf is easily hydrolyzed. Previous works revealed that the main component of natural fibers decomposes at different temperature ranges. Hemicellulose decomposes between 220 and 315 °C, cellulose at 315–400 °C, and lignin degradation covers the entire temperature range [54,55,56]. The high crystallinity of cellulose resulted in a higher decomposition temperature compared to hemicellulose. However, as for lignin, it is different from hemicellulose and cellulose, which is a highly branched polymer consisting of polysaccharides that are responsible for the higher decomposition temperature of the lignin [57].

Table 3 shows the degradation parameters of thermogravimetry analysis. The elevated weight loss was recorded within the temperature range of 300–400 °C. The degradation of PLA also occurs at this temperature range [58]. Based on Figure 3, it can be observed that there are two distinct decomposition stages of the laminated composites. The first stage is between 250 and 400 °C of the temperature range and the second stage occurs between 400 and 470 °C. The different peak intensities that can be observed in Figure 3 are due to the material composition of the hybrid composites, as these materials degraded at different temperatures [59]. The first stage is related to the decomposition of the major component in natural fibers and PLA. While the second decomposition stage is due to the decomposition of polyester fiber at a higher temperature. Aisyah et al. [60] also reported the same thermal degradation temperature range of 220–420 °C, where a major decomposition of kenaf fiber and the degradation of polymers occurred. The decomposition of the laminated composites was completed at 600 °C, leaving char residue of 5.8–10%. Teh et al. [61] reported that the formation of the volatile oligomers has contributed to the weight loss of polyester at this temperature range. The decomposition temperature range of the polyester fiber obtained also agrees with the finding by Achagri et al. [62].

Figure 2 also depicts that the high layers of woven kenaf in the S7 sample reduced the thermal stability of the composites. However, other hybrid samples are more thermally stable than the S7 samples. The result obtained is due to the effect of hybridization with woven polyester. The same finding has been reported by Zuhudi et al. [42], who studied the impact of hybridization on the thermal properties of woven natural/synthetic fibers. The report revealed that the TGA curves demonstrated an increase in the thermal stability of the matrix with the incorporation of woven bamboo and glass fibers.

The fiber content influenced the thermal properties of the laminated composites. The S7 sample revealed the highest char yield among all samples. High char yield generally will improve the thermal resistance of the composites [63]. The high kenaf content in the S7 composites consists of more lignin which is responsible for the high char yield [64,65,66]. Moreover, in terms of decomposition temperature, the S8 sample exhibited the highest decomposition temperature (T_max_); thus, revealing the most thermally stable samples. However, as for hybrid composites, the S4 laminate depicted the most stable sample. In terms of stacking sequences, no significant effect can be observed for samples with different stacking sequences with the same fiber content for samples S1/S3 and S2/S6. The TGA curves for these composites portrayed almost the same pattern.

### 3.2. Differential Scanning Calorimetry (DSC)

**Figure 4:**
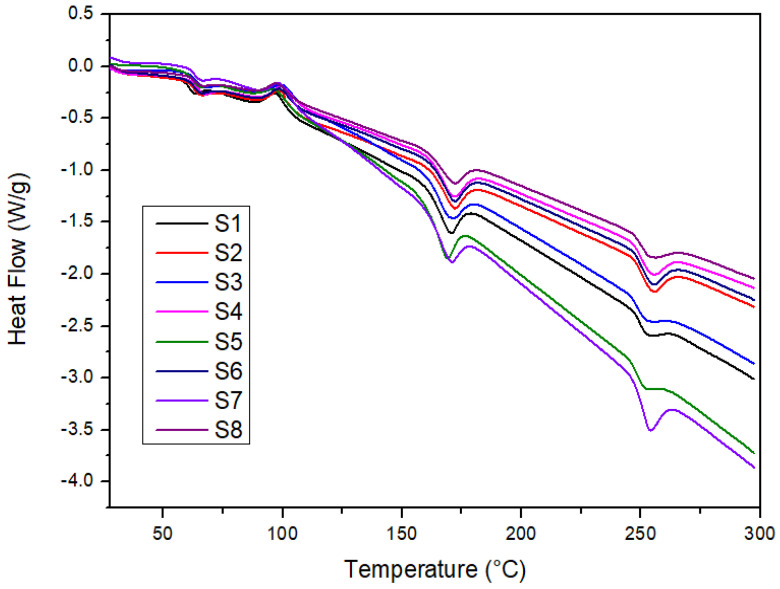
DSC thermogram of laminated composites.

The DSC analysis can further explain the thermal behavior of the laminated composites. Figure 4 shows the DSC curves of laminated composites that experienced the exothermic and endothermic processes. The DSC curves demonstrated almost the same trend for all samples in which the exothermic and endothermic processes can be identified based on multiple peaks obtained in Figure 4. Table 4 shows the differential scanning calorimetry analysis of the laminates.

The peaks in the DSC thermogram provide the glass transition temperature (T_g_), crystallization temperature (T_c_), and melting temperature (T_m_) for each sample. It can be noticed that the first transition occurs in the temperature range of 58–61 °C, indicating the glass transition temperature (T_g_) of PLA [67,68]. It can be seen that the addition of woven polyester in hybrid composites improved the glass transition temperature of all hybrid composites compared to S7. The S7 sample consists of woven kenaf and PLA only, while the S8 sample, which consists of woven polyester and PLA, recorded the highest glass transition temperature among all samples. Based on Table 3, it shows that all hybrid samples (S1, S2, S3, S4, S5, and S6) revealed a higher glass transition temperature compared to the S7 sample. The result depicted that the glass transition temperature of the hybrid laminates increased with the addition of woven polyester. The higher glass transition temperature means a higher temperature is needed to turn the sample from a glass to a rubbery state.

The continuous heating of the laminates contributed to the exothermic crystallization process known as cold crystallization, in which the transition of laminates from glassy to amorphous phase occurs [69]. At a higher temperature, the peak can be observed in the temperature range of 92–96 °C, indicating the cold crystallization temperature of PLA [70]. Iannace et al. [71] reported that the fastest cold crystallization rate of PLA occurs between 95 and 115 °C in the temperature range. Subsequently, the samples were heated until 300 °C, and two endothermic peaks can be observed. The peaks obtained are known as the melting temperature (T_m_), denoting the melting temperature of PLA and polyester. The first peak is in the range of 166–171 °C revealing the melting point (T_m_) of PLA [72,73]. Moreover, in the range of 252–257 °C, the peak obtained is due to the melting point (T_m_) of polyester [74].

### 3.3. Dynamic Mechanical ANALYSIS (DMA)

#### 3.3.1. Storage Modulus (E′)

**Figure 5:**
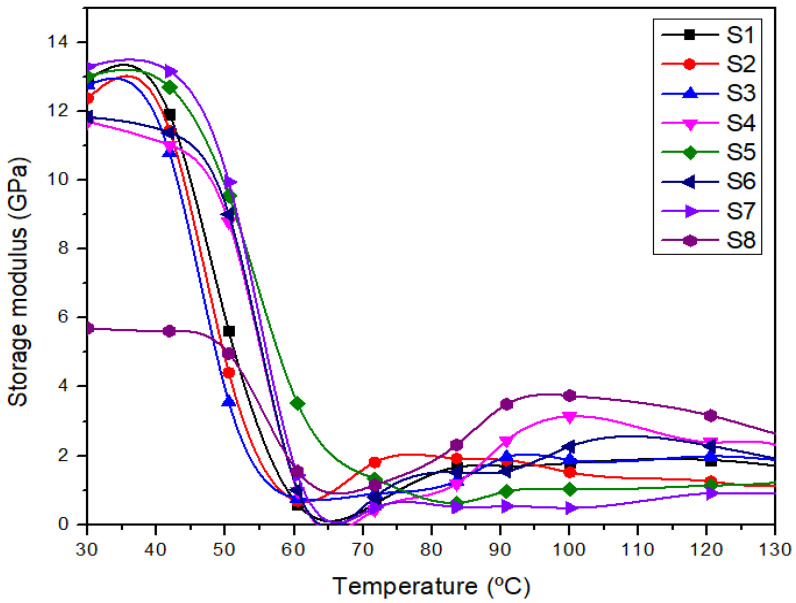
The storage modulus of different laminated composites.

Dynamic mechanical analysis (DMA) was used to measure the temperature-dependent properties of polymer composites such as storage modulus (E′), loss modulus (E″), and damping factor (Tan δ). The result gathered reflected the stiffness and damping characteristics of the laminated composites as a function of temperature. Figure 5 shows the storage modulus of different laminated composites. The storage modulus (E′) provides information regarding the rigidity, fiber-matrix adhesion, and stiffness of the composites [49,75].

Figure 5 demonstrates a variation in storage modulus (E′) of different laminated composites as a function of temperature. The storage modulus property was found to gradually decrease as the temperature increased for all laminates as a result of the reinforcement of fibers in the laminates [76,77]. Figure 5 also demonstrated the increasing trend with an increment in woven kenaf fiber loading in the following order: S8 < S4 < S6 < S2 < S3 < S1 < S5 < S7. Sathyaseelan [29], in his report, concluded that an increase in the kenaf fiber layer in composite samples made the composites stiffer and increased the storage modulus. It can be noticed that the composites experienced three phases under increasing temperature denoting the glassy, transition, and rubbery phases. These phases are a typical trend for polymer composites’ dynamic mechanical analysis curves, in agreement with the previous report [43,78]. In the glassy region, the composite structure is very tightly packed with the highest stiffness property that results in high storage modulus for all samples [78]. The tightly packed structure limited the molecular mobility of the composites; thus, contributing to the high stiffness property [79]. It was found that the S7 sample possessed the highest storage modulus, and the S8 sample consisting of all five layers of woven polyester in composite showed the lowest storage modulus.

Among hybrid composites, the S5 sample revealed the most optimum sample with the highest storage modulus. Figure 5 shows that the storage modulus of all laminates dropped when passing the glass transition region (T_g_). As the temperature increased, the breakage of cross-linking between the molecular chains for the composites that occurred at higher temperatures increased the molecular mobility of the composites. Generally, samples with high woven kenaf content show a higher storage modulus value than others. Khan et al. [44] revealed that the existence of natural fibers in hybrid composites had increased the storage modulus of the composites. The results revealed that the S7 and S5 samples that consisted of 5 and 4 layers of woven kenaf exhibited a higher storage modulus compared to others.

More woven kenaf in the hybrid composites made the composite stiffer and revealed a higher storage modulus. Moreover, the low storage modulus of the S8 sample is probably due to the high elasticity properties of woven polyester fiber that cause a low resistance to deformation [20]. The result also is in line with Nurazzi et al., who reported that pure polyester demonstrated the lowest storage modulus of composites compared to others [75].

The storage modulus was found to be decreased in the second and third regions due to the higher molecular mobility. A significant fall in curves can be observed in Figure 5 due to the increasing movement of the polymeric chain that affects the fiber-matrix adhesion and stiffness property of all composites [80]. However, the S2/S6 and S1/S3 composites with the same fiber content but different stacking sequences showed that the S6 and S1 exhibited slightly higher storage modulus values. Both composites used woven kenaf as the outer layer of the composites. The effect of different stacking sequences for laminated composites with the same fiber content seems to not significantly affect the storage modulus property.

#### 3.3.2. Loss Modulus (E″)

**Figure 6:**
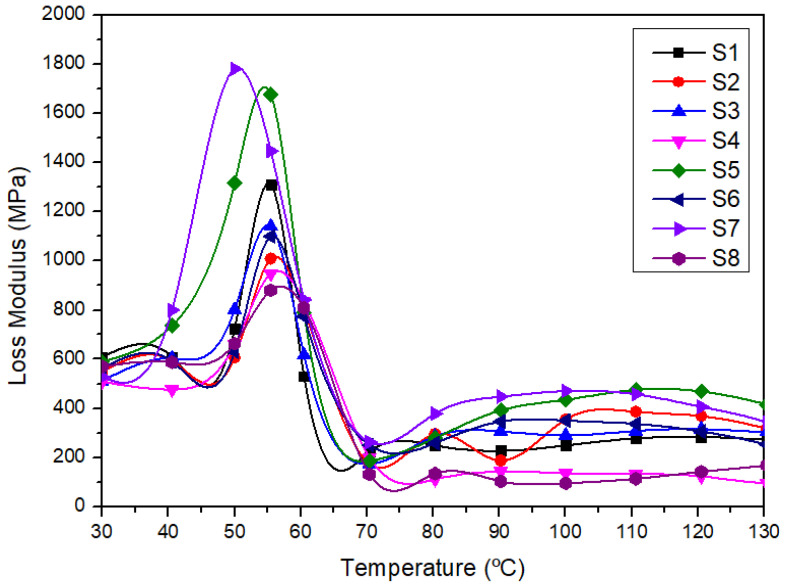
The loss modulus of different laminated composites.

The loss modulus represents the energy dissipation as heat per cycle of sinusoidal deformation because of the viscose motions inside the material. The loss modulus is maximum at the temperature, which shows that the maximum heat dissipation occurred [81]. Figure 6 shows the loss modulus of different laminated composites.

As the increasing temperature approached the glass transition temperature of each laminated composite, the molecular segmental motion was initiated [43]. The peak height of loss modulus in Figure 6 indicates the glass transition temperature (T_g_) of the polymeric system [82]. The S7 and S5 laminated composites exhibited a high loss modulus compared to other composites. The finding was supported by Akil et al. [83], who reported the same trend where the loss modulus peak increased with the increase in kenaf fiber content. The peak of loss modulus is related to the stiffness of the material and signifies the dissipation of heat energy [84]. The high loss modulus obtained is due to the increase in internal friction in the composites and contributes to higher energy dissipation [80,85]. The T_g_ for all laminates was recorded in the temperature range of 45–60 °C. The incorporation of woven polyester content in the hybrid composites shifted the transition temperature composites higher than S7, consisting of woven kenaf and PLA only.

The S8 sample recorded the lowest loss modulus value compared to others. The lower loss modulus for the S8 sample shows that without woven kenaf reinforcement, the composites become more mobile, and the finding is in agreement with what has been reported by Haris et al. [86]. However, as for hybrid composites, the S5 sample revealed the highest loss modulus compared to other hybrid composites due to the higher woven kenaf content. The higher loss modulus property for the S7 sample compared to the other composites is due to the higher woven kenaf layers in hybrid composites; thus, reducing the mobility of the matrix molecules of the hybrid laminated composites [87].

#### 3.3.3. Damping (Tan δ)

**Figure 7:**
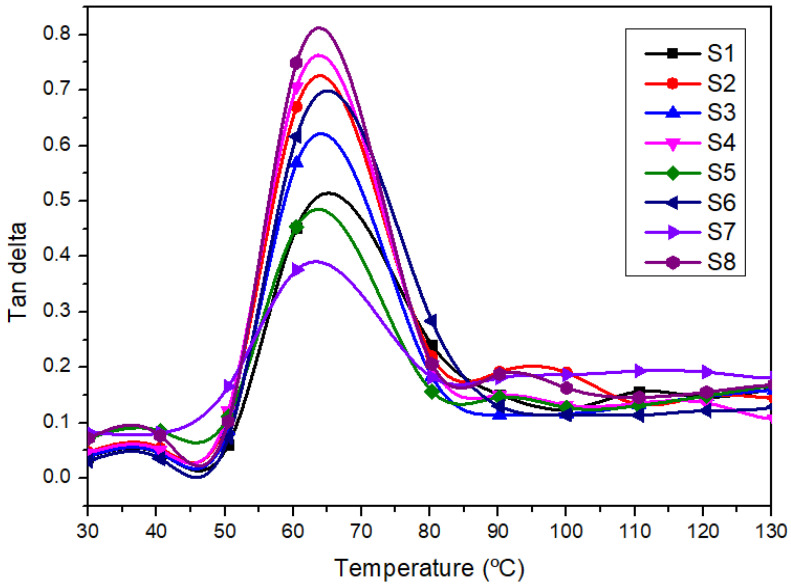
The Tan δ curves of different laminated composites.

Figure 7 demonstrates the Tan δ curves of different laminated composites. The energy dissipation behavior of the material under deformation is known as the damping factor.

Figure 7 depicts the increase in the damping factor and demonstrates multiple peaks in the transition region and then the drop in the rubbery region. An increment in Tan δ can be observed with the reduced woven kenaf in the laminated composites. The higher the woven fiber content, the lower the value of the damping peak (Tan δ). Jawaid and Khalil [88] revealed that incorporating natural fibers in the composite polymeric system would influence the damping properties. The same damping characteristic also has been reported by several researchers [29,44]. Composites with higher woven polyester content (S8 and S4) show a higher Tan δ peak than samples S1, S3, and S5. The lower Tan δ of the hybrid composites is due to the stiffness property of the hybrid composites as the addition of more woven kenaf has restricted the mobility of the polymer molecules [89]. Lee et al., 2021, in their review concluded that the restriction of the polymer chain mobility is due to the good interfacial bonding between plant-fiber-reinforced composites [90]. The same finding by Ho et al. [91] concluded that the interaction between fiber-polymer composites had restricted the polymer chain mobility of the composites. The lower Tan δ peak in the graph reveals a good interfacial adhesion between fiber reinforcement and matrix [92,93], while the higher peak indicates the lower fiber/matrix adhesion [94]. The finding shows that the improved fiber/matrix adhesion of the laminates affected the reduction of composites, in agreement with the previous works [75,93].

### 3.4. Flammability Test

**Figure 8:**
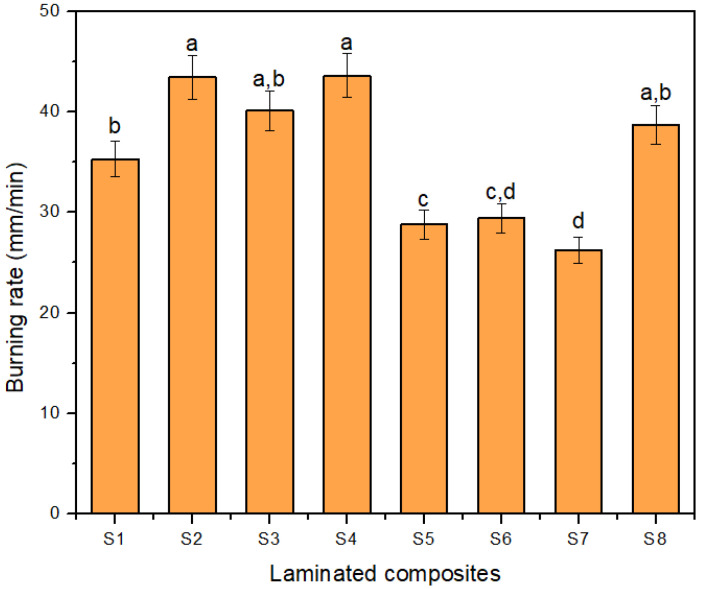
The burning rate of laminated composites. Note: Different lowercase letters indicate a significant difference.

The average burning rates of the composites measured by the horizontal burning test are shown in Figure 8. The S4 composite showed the highest burning rate compared to other samples. The highest burning rate of the S4 sample is due to the high polyester composition among hybrid composites, which indicated that the sample had the highest sensitivity to flame. The S4 sample had poor flammability behavior due to the fast thermal degradation of polymers during burning [95]. Figure 8 also revealed that hybrid samples S2, S3, and S4 with all outer layers of woven polyester displayed the top three highest burning rates.

On the other hand, the burning rate of the S7 sample revealed the lowest average burning rate. However, as for hybrid composites, the S5 sample consists of 31.7% of kenaf fiber, and the result showed that increased kenaf fiber content in hybrid composites exhibited a lower burning rate. The finding agrees with the finding by Karunakaran et al. [96], that concluded that the burning rate was decreasing with increasing kenaf fiber content. The result also revealed that the composites with the outer layer of woven kenaf (S1, S5, and S6) slowed down the burning rate of the composites. The situation is possibly due to the char formation by woven kenaf that shields the layers from being penetrated by heat and volatiles into the inner layer of the composites [96]. The char formation happened when the flame was applied toward the composites that caused incomplete combustion. The charring process removed oxygen and hydrogen; thus, the carbon remained in the char [97]. Table 5 shows the combustibility of the composite properties of the laminated composites. The S7 sample with 40% (wt.) of woven kenaf content recorded the longest time needed for the flame to reach the 100 mm mark due to the highest char formation compared to others.

Bar et al. [98] and Subasinghe et al. [99] suggested that the char layer act as a physical barrier and protection layer to the composites. The results exhibited the effect of fiber content and stacking sequences that have influenced the flammability properties of the hybrid composites.

## 4. Conclusions

The results gathered showed that the hybridization of woven kenaf and woven polyester can improve the dynamic, thermal, and flammability properties of the hybrid composites. In particular, the thermal results demonstrated the effect of the hybridization of the composites on the thermal stability and viscoelastic properties of the laminates. The work also measured the burning rate of the hybrid composites during the flammability test. The S7 sample that consisted of all woven kenaf layers in composite recorded the highest char residue of 10%, and the S8 sample displayed the highest decomposition temperature among all samples. However, as for hybrid composites, the S5 sample shows the optimum result with a high char yield and exhibited the lowest burning rate at 29 mm/min. The result revealed that the addition of woven kenaf was responsible for high char yield and prolonged the burning time of the laminates. The S5 sample also shows the optimum viscoelastic properties such as storage and loss modulus among hybrid composites.

## Figures and Tables

**Table 1:** Chemical composition of woven kenaf.

	Content	Percentage (%)
1	Hemicellulose	21.75
2	Lignin	3.29
3	Cellulose	68.48

**Table 2:** Fiber matrix ratio and thickness of the laminated composites.

		Weight (%) of Constituent			
Code	Laminate Configuration	Woven Polyester (P)	Woven Kenaf (K)	PLA	Thickness (mm)
S1	K/P/K/P/K	16.5	23.5	60	2.97 ± 0.05 ^b^
S2	P/K/P/K/P	24.5	15.5	60	2.93 ± 0.04 ^b^
S3	P/K/K/K/P	16.5	23.5	60	2.85 ± 0.01 ^a^
S4	P/P/K/P/P	32.3	7.7	60	3.06 ± 0.05 ^d^
S5	K/K/P/K/K	8.3	31.7	60	2.94 ± 0.05 ^b^
S6	K/P/P/P/K	24.5	15.5	60	3.01 ± 0.01 ^c^
S7	K/K/K/K/K	0	40	60	2.85 ± 0.03 ^a^
S8	P/P/P/P/P	40	0	60	2.84 ± 0.02 ^a^

Values with different letters in the figures are significantly different (p < 0.05).

**Table 3:** The transition temperature at 5% weight loss (T_5%_), initial degradation temperature (T_max1_), major degradation temperature (T_max2_), final degradation temperature (T_max3_), and residue of laminated composites.

	Code	T_5%_ (wt.%)	T_max1_ (°C)	T_max2_ (°C)	T_maxt3_ (°C)	Residue (wt.%)
1	S1	318.7	97.2	362.8	431.2	8.3
2	S2	327.2	93.7	362.4	432.8	7.9
3	S3	317.1	96.8	363.1	431.5	7.7
4	S4	330.2	91.2	366.7	437.7	7.1
5	S5	316.8	98.3	359.4	427.3	9.6
6	S6	326.8	94.1	364.9	433.1	8.2
7	S7	314.3	106.8	358.8	426.1	10.0
8	S8	337.5	91.4	369.8	438.2	5.8

**Table 4:** The values of glass transition temperature (T_g)_, melting temperature (T_m_), and the cold crystallization peak temperatures (T_c_) of laminated composites.

	Code	T_g_ (°C)	T_m_ (°C)	T_c_ (°C)
1	S1	58.79	93.15	168.80
2	S2	59.83	93.80	169.12
3	S3	59.21	93.27	169.09
4	S4	59.92	96.00	169.54
5	S5	58.90	95.49	168.17
6	S6	59.58	94.71	168.95
7	S7	58.51	92.65	167.92
8	S8	60.19	96.70	170.45

**Table 5:** The UL94 combustibility properties of the laminated composites.

	Code	Time for the Flame Front to Reach 100 mm Mark (sec)	Remarks
1	S1	127.5 ± 12.859 *	Fully burnt
2	S2	103.6 ± 3.067 *	Fully burnt
3	S3	112.2 ± 10.256 *	Fully burnt
4	S4	103.2 ± 0.322 *	Fully burnt
5	S5	156.2 ± 7.491 *	Fully burnt
6	S6	152.9 ± 12.693 *	Fully burnt
7	S7	171.4 ± 7.407 *	Fully burnt
8	S8	116.3 ± 13.850 *	Fully burnt

* Note: Results expressed as mean ± standard deviation.

## Data Availability

Not applicable.
